# Good agreement between questionnaire and administrative databases for health care use and costs in patients with osteoarthritis

**DOI:** 10.1186/1471-2288-11-45

**Published:** 2011-04-13

**Authors:** Daniel Pinto, M Clare Robertson, Paul Hansen, J Haxby Abbott

**Affiliations:** 1 Centre for Physiotherapy Research, School of Physiotherapy, University of Otago, Dunedin 9054, New Zealand; 2 Physical Therapy and Human Movement Sciences, Feinberg School of Medicine, Northwestern University, Chicago, IL 60611, USA; 3 Department of Medicine, Dunedin School of Medicine, University of Otago, Dunedin 9054, New Zealand; 4 Department of Economics, University of Otago, Dunedin 9054, New Zealand; 5 Department of Surgical Sciences, Dunedin School of Medicine, University of Otago, Dunedin 9054, New Zealand

## Abstract

**Background:**

Estimating costs is essential to the economic analysis of health care programs. Health care costs are often captured from administrative databases or by patient report. Administrative records only provide a partial representation of health care costs and have additional limitations. Patient-completed questionnaires may allow a broader representation of health care costs; however the validity and feasibility of such methods have not been firmly established. This study was conducted to assess the validity and feasibility of using a patient-completed questionnaire to capture health care use and costs for patients with osteoarthritis, and to compare the research costs of the data-capture methods.

**Methods:**

We designed a patient questionnaire and applied it in a clinical trial. We captured equivalent data from four administrative databases. We evaluated aspects of the questionnaire's validity using sensitivity and specificity, Lin's concordance correlation coefficient (*ρ*_c_), and Bland-Altman comparisons.

**Results:**

The questionnaire's response rate was 89%. Acceptable sensitivity and specificity levels were found for all types of health care use. The numbers of visits and the majority of medications reported by patients were in agreement with the database-derived estimates (*ρ*_c _> 0.40). Total cost estimates from the questionnaire agreed with those from the databases. Patient-reported co-payments agreed with administrative records with respect to GP office transactions, but not pharmaceutical co-payments. Research costs for the questionnaire-based method were less than one-third of the costs for the databases method.

**Conclusion:**

A patient-completed questionnaire is feasible for capturing health care use and costs for patients with osteoarthritis, and data collected using it mostly agree with administrative databases. Caution should be exercised when applying unit costs and collecting co-payment data.

## Background

The estimation of costs is an essential component in economic analyses of health care. Such estimations involve identifying the relevant cost items, determining the quantities of resources used, and assigning unit costs (also known as price weights) to the cost items [1]. Once relevant costs are identified, the types and quantities of resources used can be obtained by accessing information from health care providers, funders, or patients [2].

Providers' administrative databases and medical records are potentially rich sources of data. Auditing medical records has been considered the gold standard for identifying and quantifying health care use, but this approach has limitations [3,4]. Usually only a partial representation of direct costs is captured per provider, multiple providers must be approached for each patient, and cooperation from administrative staff is required [4,5]. Providers may restrict access to medical records due to privacy or security concerns, and computer databases may be designed to track patients' billing rather than their medical histories [4]. Medical reports may also contain inaccuracies; Jordan et al. suggests that repeat consultations and consultations for multiple conditions may not be accurately recorded [6]. Orrico reviewed discrepancies between patient self-reporting and medical records with respect to outpatient medication use and found that discrepancies arose from medical system errors in 49% of such instances [7].

When estimating costs, using the societal perspective allows for the broadest capture of costs [8]. It is also generally the 'gold standard' with regards capturing cost transfers, opportunity costs, and comparability of results [9]. In many situations patients must be asked about their health care use, expenses and other consequences, because administrative databases cannot capture all such information [10]. Methods for collecting patient-reported data include cost diaries, questionnaires, and patient interviews. These measures can be differentiated by their use of prospective versus retrospective reporting. Diaries are regarded as prospective instruments because a patient has the diary prior to the health visit and typically uses very small recall periods to record health care use, which minimizes recall bias [11]. Though the health diary has clear advantages when capturing data about daily symptoms, the superiority of this method for capturing health care use is less clear [11]. Major disadvantages of diaries include the burden on patients, complexity of data processing and analysis, exclusion of patients who are functionally illiterate, inability of researchers to immediately probe for additional data, high levels of item-level missing data, and dependence on participants' motivation to record their data [11,12]. Retrospective methods for capturing health data include questionnaires and patient interviews. Like diaries, these methods can provide researchers with ample detail and information unavailable from provider databases; however, because they are retrospective they are susceptible to recall bias. The risk of recall bias can be reduced by carefully designing the questionnaire or interview instrument [13].

It is important before applying a self-report instrument in an economic study to assess its accuracy [14]. In this study we describe the development and testing of the Osteoarthritis Cost and Consequences Questionnaire (OCC-Q) for capturing health care use and costs in patients with osteoarthritis. Our primary objectives were to assess the validity and feasibility, in a clinical trial setting, of using the OCC-Q compared with using administrative databases and to compare the research costs of these two data-capture methods.

### The New Zealand health system context

New Zealand's health system is a public system, mainly funded from taxation. Most hospitals are publicly owned and are administered by 21 District Health Boards (DHBs) throughout the country. Along with community guidance, primary health care is managed by DHBs and Primary Health Organisations (PHOs), whose job it is to provide primary health care services to enrolled patients. PHOs include multiple primary health care providers including general practitioners (GPs), nurses, pharmacists, and physiotherapists [15]. The government funds PHOs via DHBs through weighted capitation where funding rates increase based on the expected number of primary health visits per annum and the specified range of health care needs of the enrolled patient [16-18]. Individuals must register with PHOs in order to receive subsidised primary care [15]. Services provided by medical specialists and allied health workers are mostly free to patients if the services are provided via DHBs; otherwise patients usually incur the full price as out-of-pocket costs [17]. Publicly-funded pharmaceuticals are purchased by New Zealand's Pharmaceutical Management Agency (PHARMAC), and most physician-prescribed pharmaceuticals are available to patients for a $3 co-payment.

## Methods

Data intended for performing a cost-utility analysis were collected as part of the Management of Osteoarthritis (MOA) trial - a randomized controlled trial investigating the value of physiotherapy in addition to usual care for patients with hip or knee osteoarthritis (OA) undertaken in Dunedin, New Zealand [19]. Participants were recruited at the Dunedin Hospital Orthopaedic Clinic and from GP clinics in the local metropolitan area. Research nurses screened potential participants by reviewing patients' charts and interviewing them by telephone. Patients were included in the trial if they met clinical criteria for hip or knee OA according to the American College of Rheumatology [20,21]. They were excluded if they had any of the following: a joint replacement in the affected joint; any surgical procedure of the lower limbs in the previous 6 months; rheumatoid arthritis; initiation of an opiate analgesic, corticosteroid, or analgesic injection within the previous 30 days; uncontrolled hypertension or moderate-to-high risk of cardiac complications during exercise; other physical impairments precluding safe participation in the trial; an inability to comprehend instructions; and an inability to complete the trial [19]. Participants gave their informed consent to allow their medical records to be reviewed and compared, and ethical approval was granted by the Lower South Regional Ethics Committee.

### Questionnaire development

The OCC-Q (the Osteoarthritis Cost and Consequences Questionnaire) was designed to capture health care use, patient co-payments, and other out-of-pocket costs related to hip or knee OA over the preceding three-month period. We developed the questionnaire based on existing tools and recommendations [22-24] with input from experts in public health, health economics, clinical pharmacy and physiotherapy. Many of the questionnaire sections were modeled after the questionnaire reported by Cochrane et al [22]. Modifications included the standardization of the recall timeframe to three-months for the majority of health care use and increased detail of resource utilization domains. Health care services and expenses with the potential to contribute significantly to OA-related costs such as OA-related medications and inpatient care were listed in detail to improve participants' recall and decrease the effect of fatigue on recall [13,23]. The questionnaire was assessed to ensure that resource utilization domains, recommended for the assessment of costs in musculoskeletal diseases, were being captured [24]. The questionnaire was pilot-tested on patients with OA to check the clarity of its questions and its capacity to capture OA-related costs and consequences.

At the three-month follow-up point (April 2008 to December 2009), a cover letter and questionnaire with a return postage-paid envelope were sent to the first 56 participants in the MOA trial. The cover letter explained our interest in the participant's health care use since the start of the MOA trial, with each participant's trial start date mentioned to improve recall. The questionnaire asked the participant to recall his or her visits to GPs, public and private hospitals, and any community services received. It also asked about any time off work, co-payments or out-of-pocket costs related to OA over the three-month period, and use of OA-related medications during the previous week. Participants were invited in the cover letter to ring the contact phone number provided if they had any questions. The cover letter also mentioned that a researcher would phone to conduct a brief interview after the questionnaire was returned. If questionnaires were not received by one week following the mailing, participants were phoned and reminded about the questionnaire; and if necessary, this was repeated after two weeks.

The first author (DP) followed-up the returned questionnaires with a 5-10 minute phone call to each respondent to review his or her responses, in particular, with respect to the study's definition of OA-related health care. Respondents were encouraged to define a GP or hospital visit as OA-related if it was a follow-up for their hip or knee complaints, if a significant part of the visit was devoted to their hip or knee complaints, or if the doctor renewed their OA-related prescriptions. OA-related medications were specified as a pre-defined list of analgesics, anti-ulcerants, and psychotropics in the questionnaire. Interview techniques including prompting, and relating health care consultations to a consequence, such as a prescription or referral, were used to improve recall [6,13].

### Administrative databases

Covering the same three-month period as the questionnaire, the lead author (DP) collected data on each participant's use of GP services, hospital services, and medication use from administrative databases. GP contacts, which included visits to the GP or practice nurse and any contact for a renewal of OA-related medications, were captured by: (1) performing electronic database queries in GP offices using the Medtech patient management system (Medtech Global Ltd); and (2) by manually searching medical records at 17 GP offices in the Dunedin metropolitan area. Patient co-payments were captured from the GP record. Three medical records from one additional GP office were obtained via a telephone interview with the GP. A query of the local hospital's medical records captured data on each participant's use of OA-related inpatient and outpatient services by the sole public provider, the Otago DHB. Data on all medical imaging services were captured from the EasyRIS database (Philips Medical Systems) operated by the Otago DHB. A query of the New Zealand Health Information Service (NZHIS) database captured data on dispensed medications funded by PHARMAC (New Zealand's Pharmaceutical Management Agency). The same definition of OA-related health care as expressed above was used to screen the administrative databases; likewise, only OA-related medications listed in the questionnaire were included in the NZHIS query. Medication costs, quantities dispensed, and expected patient contributions were captured from the NZHIS query.

### Price weights

All costs in this study are reported in 2009 New Zealand dollars (NZD); the conversion rate to US dollars (USD) at 2009 purchasing power parity is $1 NZD=$0.63 USD. All costs are exclusive of government Goods and Services Tax (GST) in order to represent costs to society. We applied the price weights reported in Table 1 to the questionnaire results. Participants were asked to report out-of-pocket costs for health care including GP contacts and pharmaceutical prescriptions to enable the assessment of agreement between the methods of reporting with respect to out-of-pocket costs. For simplicity, hereinafter we refer to all patient out-of-pocket costs as co-payments. The NZHIS query reported the government subsidy for each relevant medication and an expected co-payment. To generate an estimate of a comparable subsidy using the OCC-Q, self-reported daily quantities of medications were extrapolated to three-month quantities, and then multiplied by the appropriate medication unit cost as reported in the New Zealand Pharmaceutical Schedule [25]. Consistent with prescribing of PHARMAC-funded medications in New Zealand, all prescriptions were assumed to provide three months' supply except for Class B controlled drugs, which are prescribed for just one month [25].

**Table 1:** Price weights for the estimation of costs*

Cost items	Price weight	Source
**General practitioner (GP) services**		
GP visits	$62	Average private fee from 10 Dunedin (New Zealand) GP offices
Prescription	$12	
**Medications**		
Analgesics	Price weight based on	New Zealand Pharmaceutical Schedule
Anti-ulcerants	medication dosage and number	
Psychotropics	of tablets/capsules issued	
**Hospital costs**		
Inpatient services	$3,983.33 multiplied by inpatient service case weights	New Zealand's case-mix framework for publically funded hospitals for fiscal year 2008/09: WIESNZ08
Emergency visits	$322	Hospital's volume schedule from funder
Outpatient orthopaedic visits	$158	
Rheumatologist visits	$210	
Medical imaging	$55 multiplied by relative value unit of procedure	Imaging contract between the Accident Compensation Corporation and the hospital

*All monetary values are in 2009 New Zealand dollars (NZD); the conversion rate to US dollars (USD) at 2009 purchasing power parity is $1 NZD = $0.63 USD.

### Item-level missing values

Missing data were imputed using ICE (imputation using chained equations) in Stata version 11.1 (Stata, College Station, Tex., USA) [26] for each missing item of resource use versus total cost. ICE was chosen because it provides support for categorical missing values, using logistic regression for binary variables and multinomial or ordered logistic regression for categorical variables [27].

### Data analysis

We measured the agreement between the estimates of each participant's health care use as captured by the OCC-Q and the administrative databases. The administrative database records served as the gold standard to compute sensitivity and specificity. Sensitivity is the proportion of participants who reported health care use when the administrative database indicates that such services were used. Specificity is the proportion of participants who reported no health care use when the database record indicates that no services were used. Sensitivity and specificity were calculated to assess agreement for the dichotomous (yes/no) reporting of health care use with respect to GP contacts, individual medication categories, inpatient services, outpatient orthopaedic visits, outpatient rheumatology visits, and medical imaging procedures.

To assess the level of agreement between the reported quantities and costs of health care use, we used Lin's concordance correlation coefficient (*ρ*_c_) which measures agreement between two continuous variables taking into account systematic bias [28]. Ranging between 1 (perfect agreement) and -1 (perfect inverse agreement), *ρ*_c _represents the extent to which the compared data deviate significantly from perfect concordance [29]. A significant advantage for our analysis is that *ρ*_c _can be applied to data from non-normal distributions, such as cost data. Values for *ρ*_c _were interpreted according to these levels of agreement: 'poor' = 0.00-0.40, 'fair' = 0.40-0.59, 'good' = 0.60-0.74 and 'excellent' = 0.75-1.00 [30]. A threshold of *ρ*_c _= 0.40 was used to determine whether levels of agreement had clinical/practical significance [30]. We also reported mean differences; 95% confidence intervals and 95% limits of agreement were reported to assess systematic bias and to identify random variation between individual measurements respectively [31,32].

With respect to missing data, we imputed 5 datasets to reflect the uncertainty surrounding the imputed values. Where mean estimates were reported, we used the estimate of the mean across the 5 imputed datasets. We performed *ρ*_c_, Bland-Altman comparisons, and 95% limits of agreement using each imputed dataset. The results of all comparisons fell within the confidence intervals calculated using the other four imputed datasets, therefore we reported the estimates using the first imputed dataset only. We used Stata version 11.1 for data analysis.

## Results

Fifty of the first 56 participants in the MOA trial who were invited to participate in the present study agreed (response rate = 89%). Their average age was 70.0 years (SD ± 7.9 years), 97% identified themselves as being of Pakeha/New Zealand European ethnicity, 58% were female, 62% had a primary complaint of knee OA (38% primary hip OA), 68% had hip or knee symptoms for 3 years or more, and 68% had at least one co-morbidity. Table 2 reports the summary statistics including inter-quartile range and mean for cost comparisons. The patient interview took an average of 8 minutes. Only 1.6% of the data were missing.

**Table 2:** Summary statistics of cost comparisons between the OCC-Q and the administrative database results*

	Minimum	0.25	Median	Mean	0.75	Maximum
**General practitioner costs**						
OCC-Q	0	62	62	80.08	124	248
General practitioner database	0	62	62	71.80	86	310
**Medication costs**						
OCC-Q†	0	9.91	26.51	34.30	48.94	210.12
New Zealand Health Information System	0	3.22	21.22	36.79	50.8	305.14
**Hospital costs**						
OCC-Q	0	0	37.74	393.53	233	14,599.83
Otago District Health Board query	0	0	0	376.87	157.52	14,599.83
**Total costs**						
OCC-Q†	0	88.49	219.99	516.52	326.63	15,141.16
Databases	0	72.59	144.78	485.46	243.40	15,029.33

*All monetary values are in 2009 New Zealand dollars (NZD); the conversion rate to US dollars (USD) at 2009 purchasing power parity is $1 NZD = $0.63 USD.

† The first imputed dataset was used for descriptive statistics

Abbreviations: OCC-Q, Osteoarthritis Cost and Consequences Questionnaire.

### Validity

#### Agreement for dichotomous reporting

As reported in Table 3, agreement for the dichotomous reporting of health care use varied. Generally, a trade-off was apparent between values for sensitivity and specificity. The self-reporting of GP contacts, paracetamol, NSAIDs, and rheumatology had higher levels of sensitivity (93%-100%) than specificity (63%-100%). The self-reporting of opiates, paracetamol and opiate-combination medications, anti-ulcerants and medical imaging had higher specificity (93%-100%) than sensitivity (44%-80%).

**Table 3:** Cross tables, sensitivity, and specificity for the number of patients using health care as self-reported and from administrative databases

Health care				Sensitivity	Specificity
**General practitioner**					
GP contacts		YES (PT)	NO (PT)		
	YES (DB)	38	3	93%	56%
	NO (DB)	4	5		
**Medications**					
Paracetamol		YES (PT)	NO (PT)		
	YES (DB)	23	0	100%	59%
	NO (DB)	11	16		
Opiates		YES (PT)	NO (PT)		
	YES (DB)	4	5	44%	93%
	NO (DB)	3	38		
Paracetamol & opiates*		YES (PT)	NO (PT)		
	YES (DB)	4	5	44%	93%
	NO (DB)	3	38		
NSAIDs		YES (PT)	NO (PT)		
	YES (DB)	17	1	94%	84%
	NO (DB)	5	27		
Pantoprazole		YES (PT)	NO (PT)		
	YES (DB)	2	1	67%	100%
	NO (DB)	0	47		
Omeprazole		YES (PT)	NO (PT)		
	YES (DB)	12	3	80%	91%
	NO (DB)	3	32		
Amitriptyline		YES (PT)	NO (PT)		
	YES (DB)	2	1	67%	98%
	NO (DB)	1	46		
**Hospital services**					
Emergency visits		YES (PT)	NO (PT)		
	YES (DB)	2	0	100%	100%
	NO (DB)	0	48		
OA-related inpatient events		YES (PT)	NO (PT)		
	YES (DB)	2	0	100%	100%
	NO (DB)	0	48		
					
Orthopaedics		YES (PT)	NO (PT)		
	YES (DB)	7	3	70%	85%
	NO (DB)	6	34		
Rheumatology		YES (PT)	NO (PT)		
	YES (DB)	2	0	100%	98%
	NO (DB)	1	47		
Medical imaging services†		YES (PT)	NO (PT)		
	YES (DB)	10	4	71%	81%
	NO (DB)	7	29		

*Combination drug

†n = 48

*Abbreviations: *GP, general practitioner; DB, database; PT, patient; Opiates=codeine, DHC continus; NSAIDs, non-steroidal anti-inflammatory drug = diclofenac, voltaren, ibuprofen, naproxen

#### Agreement for quantity reporting

Only GP contacts and medications were recorded in sufficient quantities for their quantity data to be analysed using Lin's *ρ*_c _and Bland-Altman comparisons. As reported in Tables 3 &4, participants recounted fewer GP contacts and more medications using the OCC-Q; however the mean differences between assessment methods were small: 0.06 and -0.22 respectively. Concordance levels were fair (*ρ*_c _= 0.41) for the number of GP contacts and good (*ρ*_c _= 0.63) for the number of medications reported.

**Table 4:** Agreement between the OCC-Q and the databases for the reported quantity of health care used

	OCC-Q Mean ± SD	Databases Mean ± SD	*ρ*_c_*	Mean difference (95% CI)†	95% limits of agreement
GP contacts	1.3 ± 1.0	1.4 ± 1.2	0.41	0.06 (-0.3, 0.4)	-2.34 to 2.46
Total number of medications	1.8 ± 1.2	1.6 ± 1.2	0.63	-0.22 (-0.50, 0.07)	-2.25 to 1.8

*ρ_c _= Lin's concordance correlation coefficient

†95% CI assumes a normal distribution

*Abbreviations: *OCC-Q, Osteoarthritis Cost and Consequences Questionnaire; GP, general practitioner; SD, standard deviation; CI, confidence interval

#### Agreement for reporting of costs

Table 5 compares cost estimates derived from the OCC-Q and administrative databases respectively. Cost estimates for GP services and the majority of medication subsidies exhibited acceptable levels of agreement (*ρ*_c _≥ 0.40). The majority of co-payments for medications showed poor agreement (*ρ*_c _< 0.40), as did total medication costs. All hospital services resulted in acceptable agreement except for orthopaedics (*ρ*_c _= 0.316).

**Table 5:** Costs generated by method of reporting: agreement between the OCC-Q questionnaire and administrative databases*†

	OCC-Q ($) Mean ± SD	Databases ($) Mean ± SD	*ρ*_c_‡	Mean difference ($) (95% CI)§	95% limits of agreement ($)||
GP services					
Co-payments	37.59 ± 29.46	29.62 ± 29.53	0.502	-7.97 (-16.39, 0.46)	-67.28 to 51.35
Societal cost	79.84 ± 60.84	67.08 ± 61.38	0.502	-14.24 (-30.92, 2.44)	-131.63 to 103.15
**Medications**					

**Subsidy**					
Paracetamol n = 49	2.96 ± 2.90	3.57 ± 6.36	0.358	0.60 (-1.00, 2.21)	-10.58 to 11.78
Imputed (n = 50)	3.02 ± 2.93	3.50 ± 6.32	0.360	0.58 (-1.02, 2.13)	-10.53 to 11.64
Opiates	1.89 ± 6.36	3.83 ± 11.54	0.645	1.94 (-0.24, 4.13)	-13.45 to 17.33
Paracetamol & opiates	3.20 ± 7.16	2.84 ± 7.50	0.807	-0.36 (-1.66, 0.93)	-9.48 to 8.75
NSAID n = 45	4.12 ± 7.05	3.66 ± 7.45	0.876	-0.44 (-1.52, 0.64)	-7.63 to 6.74
Imputed (n = 50)	4.74 ± 7.60	3.59 ± 7.11	0.789	-1.10 (-2.42, 0.22)	-10.40 to 8.19
Pantoprazole	0.43 ± 2.13	0.67 ± 2.74	0.716	0.24 (-0.28, 0.77)	-3.44 to 3.92
Omeprazole n = 45	2.89 ± 6.34	17.30 ± 50.56	0.140	14.41 (0.30, 28.51)	-77.62 to 106.44
Imputed (n = 50)	3.56 ± 6.35	19.03 ± 49.32	0.137	15.45 (2.42, 28.48)	-76.24 to 107.15
Amitriptyline	0.45 ± 1.72	0.30 ± 1.27	0.225	-0.15 (-0.68, 0.36)	-3.91 to 3.61
**Co-payment**					
Paracetamol n = 40	5.08 ± 16.52	0.83 ± 1.36	-0.006	-4.25 (-9.57, 1.07)	-36.79 to 28.59
Imputed (n = 50)	8.88 ± 36.86	0.66 ± 1.26	-0.008	-12.34 (-22.55, -2.13)	-84.19 to 59.51
Opiates	0.30 ± 0.91	0.40 ± 1.18	0.716	0.10 (-1.24, 0.32)	-1.48 to 1.68
Paracetamol & opiates¶	1.39 ± 4.05	0.48 ± 1.85	0.168	-0.91 (-2.06, 0.24)	-9.00 to 7.18
NSAID n = 44	1.23 ± 4.98	0.93 ± 1.66	0.151	-0.30 (-1.77, 1.18)	-9.76 to 9.21
Imputed (n = 50)	1.18 ± 4.68	0.88 ± 1.61	0.148	-0.28 (-1.58, 1.02)	-9.41 to 8.85
Pantoprazole	0.12 ± 0.59	0.12 ± 0.59	1.00	0.00 (0.00, 0.00)	0.00 to 0.00
Omeprazole n = 44	0.82 ± 2.45	0.47 ± 1.11	0.176	-0.34 (-1.08, 0.40)	-5.22 to 4.54
Imputed (n = 50)	1.06 ± 3.31	0.42 ± 1.05	0.143	-0.54 (-1.22, 0.14)	-5.34 to 4.26
Amitriptyline	0.18 ± 0.72	0.06 ± 0.42	-0.031	-0.12 (-0.36, 0.12)	-1.81 to 1.58
**Total medication cost **n = 31	24.36 ± 25.04	41.00 ± 59.76	0.144	-16.63 (-38.49, 5.23)	-135.82 to 102.56
Imputed (n = 50)	30.40 ± 39.83	36.79 ± 52.90	0.152	5.70 (-11.51, 22.91)	-115.41 to 126.81
**Hospital-based services**					
Emergency visits	13.36 ± 66.11	13.36 ± 66.11	1.00	0.00	0.00 to 0.00
OA-related inpatient events	292.00 ± 2064.73	292.00 ± 2064.73	1.00	0.00	0.00 to 0.00
Orthopaedics	50.41 ± 80.76	37.80 ± 81.51	0.316	-9.45 (-35.68, 16.78)	-194.03 to 175.13
Rheumatology	16.76 ± 71.33	12.57 ± 65.71	0.701	-4.19 (-12.61, 4.23)	-63.45 to 55.07
Medical imaging n = 48	24.15 ± 35.57	21.13 ± 34.23	0.488	-3.02 (-12.67, 6.63)	-70.94 to 64.90
Total hospital costs	393.53 ± 2054.51	376.87 ± 2057.66	0.998	-16.66 (-48.01, 14.68)	-237.25 to 203.93
**Total health care cost **n = 31	209.27 ± 167.95	192.51 ± 183.74	0.606	-16.75 (-73.84, 40.33)	-328.01 to 294.51
Imputed (n = 50)	513.20 ± 2132.22	485.46 ± 2105.69	0.997	-34.05 (-76.65, 8.55)	-333.83 to 265.73

*All monetary values are in 2009 New Zealand dollars (NZD), with a conversion rate to US dollars (USD) at 2009 purchasing power parity of $1 NZD = $0.63 USD.

†OCC-Q datasets are complete (n = 50) unless indicated otherwise (Database records have no missing values), n < 50 = complete case analysis for OCC-Q with the same cases dropped from the Database, Imputed n = 50 indicates the mean ± SD for OCC-Q values are estimated from 5 imputed datasets combined; *ρ*c, mean difference 95% CI, and 95% limits of agreement are estimated using the first imputed dataset

‡*ρ*c = Lin's concordance correlation coefficient

§95% CI assumes a normal distribution

||95% limits of agreement

¶Combination product

*Abbreviations: *OCC-Q, Osteoarthritis Cost and Consequences Questionnaire; GP, general practitioner; SD, standard deviation; CI, confidence interval; NSAID, non-steroidal anti-inflammatory drug: diclofenac, voltaren, ibuprofen, naproxen; Opiates: codeine, DHC continus

### Research costs

Table 6 summarizes the research costs incurred for each data-collection method. The cost of the questionnaire-based method was less than one-third of the cost of extracting the same data from administrative databases, enabling a potential savings of $1,940 NZD for our sample.

**Table 6:** Incremental research costs associated with the methods used to collect health care use and cost data*

	OCC-Q	GP records	Hospital database	Medical imaging records	New Zealand Health Information System	Combined databases
	$	$	$	$	$	$
Materials (× 50)	170.04	0	0	0	0	0
• 13 page questionnaire						
• Cover letter						
• 1 Legal envelope						
• 1 Letter envelope						
• Return postage						
Printing, assembling, mailing (time)†	83.60	0	0	0	0	0
Telephone follow-up†	42.85	0	1.67	1.67	3.55	6.89
Emails†	0	0	1.67	1.67	1.67	5.01
Query design	0	0	784.58‡	150.47‡	141.22‡	1076.27
Data collection (patient level)	217.78†	262.09†	232.73§	135.23§	0	630.05
Questionnaire scanning†	51.21	0	0	0	0	0
Data normalization†	334.40	250.80	125.4	41.80	167.20	585.20
Travel reimbursement	0	176.80¶	0	0	0	176.80
Query building/reporting fee	0	160.00	0	0	200.00	360.00
Total cost	899.88	849.69	1146.01	330.84	513.64	2840.22

*All monetary values are in 2009 New Zealand dollars (NZD); the conversion rate to US dollars (USD) at 2009 purchasing power parity is $1 NZD = $0.63 USD

Abbreviations: OCC-Q, Osteoarthritis Cost and Consequences Questionnaire; GP, general practitioner

†Time valued at research assistant hourly rate of $20.90

‡Hospital database query design

§Hospital database: 7 hours of computer analyst time ($32.50), 0.25 hours research assistant time ($20.90), radiology database: 4 hours computer analyst time, 0.25 hours research assistant time

¶Travel reimbursement was $0.85/Km

## Discussion

We found that using a patient-completed questionnaire was a feasible and valid method of capturing health care use and costs for patients with OA compared with accessing administrative databases. First, with respect to the questionnaire's feasibility and research costs, response rates were high (89%); though a minority of participants needed up to two telephone calls in order to return the questionnaire. Item-level missing data were minimal (1.6%) and research costs were considerably lower than relying on administrative databases. A review by Verbrugge [11] showed a range of 7-33% of item-level missing data for diaries. This metric was not reported by Goossens et al. [10]; however they reported that only 68% of diaries were returned.

As for previous studies [3,4,10,33,34], we found high levels of agreement between the data-collection methods for salient, high-cost health services such as hospitalizations and emergency visits. Participants more accurately reported their non-use (specificity) than use of health care (sensitivity). This likely reflects the three-month time horizon, which for most participants resulted in a relatively low use of health care. For GP contacts, paracetamol, and NSAIDs, sensitivity was higher than specificity (Table 2). The lower specificity levels for GP contacts may be the result of recall bias. Given the short timeframe of our study, it is unlikely this discrepancy reflects participants forgetting their visits. Rather, it may be the result of 'telescoping' (or 'reverse telescoping') which occurs when a person includes (or 'telescopes') health care used outside of the study time period, or when health care from within the study time period is 'reverse telescoped' out [13]. Telescoping does not result in a consistent bias [13]. The lower specificity of paracetamol and NSAIDs may be associated with the lower actual use of these medications (often taken on an 'as needed' basis) than as prescribed. In other words, a participant may have accurately reported use of a medication, but because this was less than prescribed, no new dispensing of the medication was required within the three-month time horizon leaving no record in the database.

We were able to only analyse quantity data for GP visits and the number of medications used. Utilization was too low to assess quantity data for the other health care included in our study. Levels of agreement between the assessment methods were acceptable (*ρ*_c _> 0.40) for the number of GP visits and total number of medications used. We also compared cost estimates using Lin's concordance correlation coefficient (*ρ*_c_) and Bland-Altman comparisons. Societal costs and co-payments associated with GP visits were in agreement with database results (*ρ*_c _= 0.502). For the majority of medication subsidies, the cost estimates from the OCC-Q and NZHIS agreed. The questionnaire underestimated paracetamol subsidy by an average of $0.58 per person (2009 NZD) and overestimated patient co-payment by $12.34 per person. We believe this to be the result of some participants being prescribed the medication by their GP but, instead of filling the prescription at a pharmacy, they chose to purchase the medication over-the-counter.

The self-reporting of omeprazole subsidy disagreed with the NZHIS results (*ρ*_c _= 0.13) despite dichotomous reporting of omeprazole use corresponding to sensitivity and specificity levels of 80% and 91% respectively. This discrepancy appeared to be related to the reimbursement of a more expensive brand-named drug, Losec, in the NZHIS record. We did not account for the reimbursement of Losec in the questionnaire-based estimates because it was not included in the pharmaceutical schedule. We can only guess why Losec was reimbursed by the government agency (PHARMAC) despite it not being on the pharmaceutical schedule. Perhaps it was the result of a rebate agreement with the manufacturer or a purchase by the Otago DHB. The removal of omeprazole from the total medication cost increased the concordance correlation coefficient to levels well beyond the threshold of clinical/practical agreement (data not reported). This illustrates the importance of knowing which unit costs to use when estimating costs. It also shows how reasonable attempts at using the appropriate unit cost may not capture costs that are not reported in the public domain.

For most medications, there were poor agreements for co-payments, though mean differences were low (< $4.26 for all but paracetamol). This was likely due to a combination of participants not accurately recalling their co-payments and errors in the expected contribution reported by NZHIS. This was a particularly difficult task for participants because they often combined store purchases with medication co-payments when at the pharmacy. For this reason, we recommend that analysts use caution when capturing co-payments for particular cost items. An understanding of the environment in which co-payments are made is important. For example, an option other than self-report may be required for studies set in countries where medication co-payments take place in a pharmacy in which purchases unrelated to health care can be made. If available, options such as average medication co-payments may be applied. However, considering the low impact of the co-payment on the cost of total health care use, it may not be worthwhile investing a large amount of analyst resources to increase the accuracy of this particular cost [1].

Our results compare favourably with other studies that assessed agreement between patient report and administrative records [3,34]. We compared our results with two studies that reported data in cross tables, which allowed us to calculate sensitivity and specificity from their findings. Data reported by Ruof et al. indicated excellent sensitivity for physician visits (100%) [34]. This physician visit category was a general measure that included GPs and specialists. Ruof et al. grouped physicians to increase agreement; however applying costs to such a general measure would be difficult. Disaggregated physician visits were not included in the cross tables but the authors indicated that kappa values were < 0.2 (poor agreement) for all physician categories [34]. With respect to GP visits, data reported by Raiana et al. indicated sensitivity levels (95%) similar to our study (93%), but lower specificity levels (20%) suggesting that participants in their study were less able to accurately recall a non-attendance than our participants [3]. Our results were stronger for the quantity of GP visits reported when compared with Raiana et al. (intra-class correlation coefficient of 0.34), though their population had a greater proportion of demographic factors associated with poorer agreement [3]. Ruof et al. reported high sensitivity levels (> 90%) for the majority of medications assessed [34]. This was higher than for our study, which may have been due to differences in the populations studied. Ruof et al. studied patients with rheumatoid arthritis, who typically receive intensive, medication-based treatment that slows disease progression [23]. The primary use of medication as a disease-modifying treatment may increase a patient's ability to recall use of that particular health care item [13].

Two characteristics of our study may have increased the complexity of our data collection and analysis. The first is the use of an imperfect gold standard. Administrative databases have been shown to have errors [6,7]. Having an imperfect gold standard complicates the assessment of accuracy, often resulting in an upward or downward bias of the accuracy estimate [35]. Reitsma et al. review methods to correct the gold standard which include adjusting it by percentages of error that are found in the literature or that may be considered plausible [35]. However, little evidence exists to guide the correction of cost data from administrative databases. An example from our study illustrates imperfection of our gold standard with respect to the reporting of OA-related inpatient services. One total knee replacement was recorded on the patient questionnaire, but not in the administrative database. When manually searching the hospital records for this participant we found operative notes indicating that a knee replacement had indeed been performed during the study period. In addition to decreasing our estimate of accuracy for the reporting of OA-related inpatient events, the removal of this procedure from total costs of health care services would have substantially affected our results.

The second characteristic that may have complicated data collection was the use of OA-related costs. There is a possibility that patients and researchers do not agree on what 'OA-related' means. We attempted to minimize this discrepancy by using firm definitions for determining which services constitute OA-related health care. Though we used definitions for OA-related health care use, we acknowledge that medicine is multifactorial and that there is little evidence supporting the use of definitions to identify what is and is not disease-related [36]. The decision to use OA-related costs was made in order to reduce variability of our cost estimates by limiting costs to those related to OA. The results of our study may give researchers who are interested in analyses of 'OA-related' disease support in the application of a definition that resulted in high levels of agreement between patients filling out a questionnaire and researchers applying the definition against administrative data [35].

Our study has several limitations. First, we have a small sample size. We reported descriptive results so that our results are interpretable within the context of the sample, and we provided cross tables to allow comparisons with current and future studies. Second, we did not screen our participants with a mental health assessment in order to decide who should be allowed to participate in the self-reporting exercise. By not screening participants in this way, a small number of participants may not have been able to complete the questionnaire accurately; however, considering the age range of our population and features of our questionnaire designed to assist recall, we believe this to be of little consequence in our study. Third, the reduced research costs found when using the OCC-Q may have been related to the small sample size. Fixed versus variable costs differ between the analytic approaches used in the study. Many of the databases had larger fixed costs and smaller variable costs when compared with the questionnaire. For example, if the sample size increased to 150 participants, time spent on the phone would have been unchanged for the databases but would have nearly tripled for the questionnaire-based approach. It is possible, given a significantly larger sample size, that the research costs would be higher for use of the OCC-Q than for the databases. Fourth, we were unable to compare important cost items such as patients' work-related productivity losses, community-based allied health visits, and informal care. These costs were collected using the questionnaire but were unavailable from administrative databases and so they were not included in the present study.

## Conclusions

The identification and valuation of health care use is a necessary component in economic evaluations. Our study reports on a patient-completed questionnaire that demonstrates adequate accuracy and feasibility for capturing health care use and costs, and is inexpensive compared with obtaining this information from administrative databases. However, patient-reported co-payments may be inaccurate for some cost items.

## Competing interests

The authors declare that they have no competing interests.

## Authors' contributions

All authors were involved in the study design, interpretation of the data and revising the article critically for important intellectual content, and all authors read and approved the final manuscript. DP drafted the article, acquired and analyzed the data, and takes responsibility for the accuracy of the data analysis. HA was responsible for study conception.

## Pre-publication history

The pre-publication history for this paper can be accessed here:

http://www.biomedcentral.com/1471-2288/11/45/prepub
